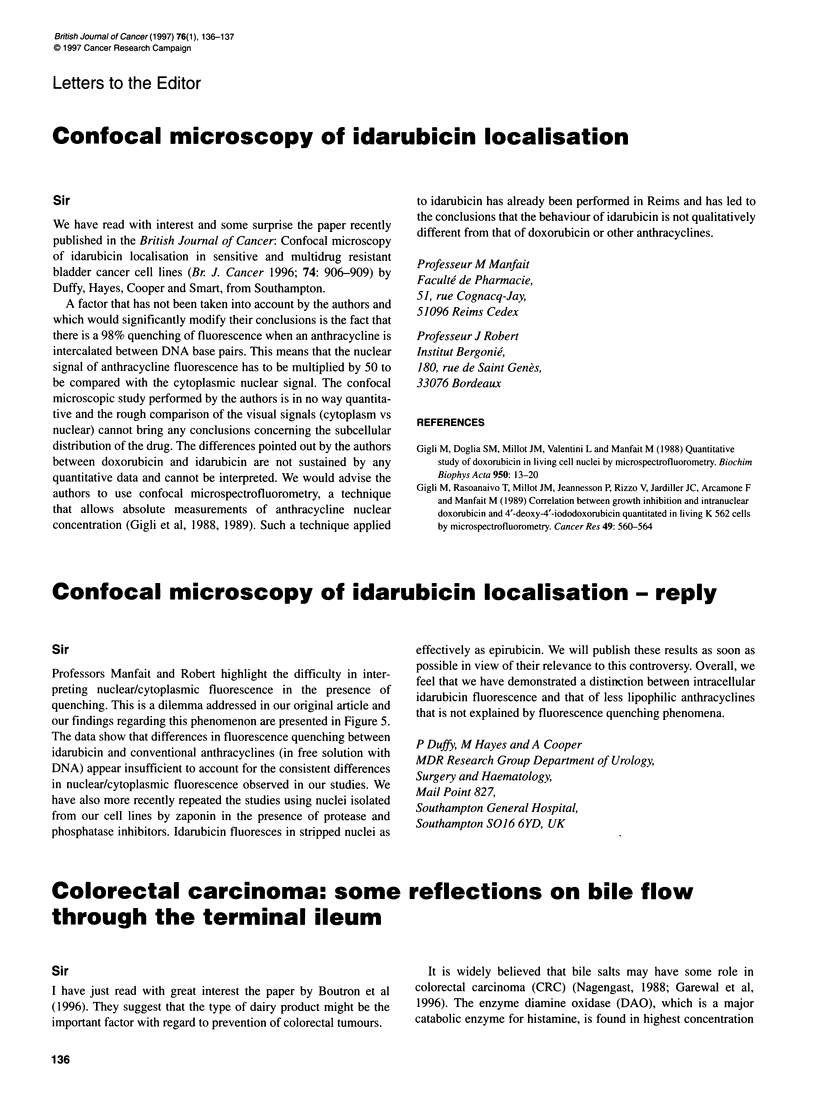# Confocal microscopy of idarubicin localisation - reply

**Published:** 1997

**Authors:** P Duffy, M Hayes, A Cooper


### Confocal microscopy of idarubicin localisation

### Confocal microscopy of idarubicin localisation – reply

### Sir

Professors Manfait and Robert highlight the difficulty in interpreting nuclear/cytoplasmic fluorescence in the presence of quenching. This is a dilemma addressed in our original article and our findings regarding this phenomenon are presented in Figure 5. The data show that differences in fluorescence quenching between idarubicin and conventional anthracyclines (in free solution with DNA) appear insufficient to account for the consistent differences in nuclear/cytoplasmic fluorescence observed in our studies. We have also more recently repeated the studies using nuclei isolated from our cell lines by zaponin in the presence of protease and phosphatase inhibitors. Idarubicin fluoresces in stripped nuclei as effectively as epirubicin. We will publish these results as soon as possible in view of their relevance to this controversy. Overall, we feel that we have demonstrated a distinction between intracellular idarubicin fluorescence and that of less lipophilic anthracyclines that is not explained by fluorescence quenching phenomena.

*P Duffy, M Hayes and A Cooper*
*MDR Research Group Department of Urology,*
*Surgery and Haematology,*
*Mail Point 827,*
*Southampton General Hospital,*
*Southampton SO16 6YD, UK*